# Household Smoking Status and Heavy Metal Concentrations in Toenails of Children

**DOI:** 10.3390/ijerph16203871

**Published:** 2019-10-12

**Authors:** Shamshad Karatela, Christin Coomarasamy, Janis Paterson, Neil I. Ward

**Affiliations:** 1 University of Queensland, Faculty of Medicine, Herston QLD 4006, Australia; 2 Ko awatea, Counties Manukau Health, Private Bag 93311, Auckland 1640, New Zealand; Christin.Coomarasamy@middlemore.co.nz; 3 AUT University, School of Public Health and Psychosocial Studies, Auckland 1640, New Zealand; Janis.paterson@aut.ac.nz; 4 University of Surrey, Department of Chemistry FEPS, Guildford, Surrey GU2 7XH, UK; n.ward@surrey.ac.uk

**Keywords:** second hand smoking, neurotoxins, child health, toenail biomarker

## Abstract

There is limited evidence on the distribution of heavy metals and its association with secondhand smoking (SHS) within Pacific Island children living in New Zealand. Certain heavy metals such as cadmium (Cd), lead (Pb), and aluminum (Al) bioaccumulates in the body and can deteriorate health in both children and adults. Others, such as chromium (Cr) and nickel (Ni) in trace amounts are necessary but become toxic at high levels. Exposure routes of these elements include food, water, and air. The purpose of this study was to identify the distribution of toxic metal concentrations and its possible correlation with SHS within the Pacific Island children. A sub-sample of children within Pacific Island families longitudinal study, at the nine-year phase, who were living in the New Zealand city of Auckland were invited to participate, (*n* = 278). Toenails were used as a biomarker to determine Cr, Pb, Cd, Cu, Ni, and Al concentration using inductively coupled plasma mass spectrometry. Reliable and validated questionnaires were used for demographics, lifestyle, and health outcome variables. Significant differences between household smoking status and ethnicity, as well as parents’ marital status, were observed (*p* < 0.05). There was no statistical difference in heavy metal concentrations in smoking versus non-smoking households. However, Cr, Pb, Cd, Cu, and Ni concentrations were all higher than the required optimal health value in both groups. A high concentration of heavy metals was observed in these children that exceeded the value required for optimal health, although no significant difference in heavy metals with regards to secondhand smoking was observed. SHS was associated with children’s ethnicity and parental marital status, but not with household income levels or maternal education.

## 1. Introduction

Exposure to secondhand smoking (SHS) in children is a serious health concern that requires further attention, as it is the most frequent indoor air pollutant [1]. SHS is the third leading cause of preventable death worldwide after smoking and alcohol, and therefore a quarter of children worldwide have been exposed to SHS [2]. Longitudinal studies have shown that maternal smoking during pregnancy had higher negative cognitive and behavioral outcomes in children [3]. Additionally, mothers who smoked one cigarette packet a day during pregnancy have children whose IQ is 2.87 points lower than children of non-smoking mothers [4]. Furthermore, mothers who are exposed to secondhand smoking have children who are more likely to have attention deficit disorder, conduct disorder, aggression, depression, and hyperactivity [5,6].

There is a growing body of evidence that demonstrates that smoking releases harmful toxicants that lead not only to adverse behavioural and cognitive effects, but also an increase in cardiovascular diseases, chronic obstructive pulmonary disease, and many forms of cancers [7,8,9]. Heavy metals can also have adverse effects on kidney function [10] as well as on the hepatic system [11]. Cigarette smoke contains toxicants that gets inhaled or absorbed by people exposed [12]. Certain heavy metals, such as chromium (Cr), lead (Pb), cadmium (Cd), copper (Cu), aluminum (Al), and nickel (Ni) have shown to be higher in smokers [13]. These metals are known to have lifelong effects on health, while others have an immediate effect, depending on the doses and type of metal [14]. For example, Pb is a risk factor for cardiovascular diseases at much lower levels and kidney damage at very high levels [15]. Furthermore, mental retardation can occur at exposures to higher levels of heavy metals and neurocognitive disorders such as impaired memory and lower IQ at much lower exposures [11]. In children, the effect of SHS is further exacerbated by the fact that they are still growing and hence absorb heavy metals at a greater rate than adults. Children whose parents smoke have double the risk of lower respiratory illnesses like bronchitis and pneumonia compared to those from parents who do not smoke [16]. Moreover, the more the parents or family members smoke around their children, the more likely these children are to have adverse health effects such as respiratory diseases, ear infections, and severe asthma attacks. Being exposed to SHS can also affect children’s development and behavior. As such, exposed children may have trouble paying attention, compromising their full potential in their learning capabilities at school [17,18].

The heavy metals can be measured in nails with a window of exposure to between 1–6 months depending on how long the nail is [19]. Metals can be deposited in the nails after being either ingested or inhaled and metabolized (within 1 to 2 months). As such, nails provide a longer integration of exposure to heavy metals [20]. Nail samples, particularly toenails, are also less exposed to external contamination [20]. Just like hair, they are simple to collect, easy to analyze, and store well [21]. Also, elements deposited into the nails are not subject to additional metabolic processes and many elements are present in the nail at substantially larger concentrations than in urine or blood [22]. There have been studies that have examined the reliability of toenail measurements [23]. Although there may be underestimation of the correlation of association with other measures due to measurement errors, the toenail concentrations of most elements are still suggested to be useful biomarkers of exposure in which a single sample is assumed to represent long-term exposure [23].

Overall, smoking rates in New Zealand (NZ) are decreasing (13% of adults smoke, as compared to 25% in 1996–1997), however, Pacific Island people still have the second highest smoking rates after Māori (23%) [24]. Exposure to SHS in the home has almost halved between 2006–2007 and 2012–2013 for New Zealand adults (7.5% to 3.7%, respectively) and for children aged 0–14 years (9.6% to 5%, respectively) [25]. There was a decline in the proportion of Pacific students exposed to smoke inside homes, from 31 percent in 2001 to 25 percent in 2006 [26]. Even though significant progress has been made in reducing the SHS exposure, non-smokers in NZ remain exposed, with the highest exposure being in pre- and school-aged children, Māori, and those of low socio–economic status [27]. A recent finding within the Pacific Island Family Cohort study at the 11-year follow-up showed a high prevalence of smoking in the fathers [28]. As such, smoking is still the leading contributor to death within the Pacific population [29].

This is the first study that aimed to explore SHS in Pacific Island children and its association with toenail heavy metals concentration. Therefore, the aim of this study was to assess the possible association between SHS and toxic metal concentrations of chromium (Cr), lead (Pb), cadmium (Cd), copper (Cu), nickel (Ni), and aluminum (Al) in nine-year-old Pacific Island children who were living in South Auckland, New Zealand. 

## 2. Materials and Methods

### 2.1. Study Area and Population

An observational study was undertaken between July 2010 and July 2011 involving a sub-sample of nine-year-old Pacific children within a Pacific Island Families (PIF) longitudinal cohort in which these individuals have been followed since birth [30]. These children were born in Middlemore hospital, South Auckland, New Zealand, to mothers in the year 2000 [30]. The eligibility criteria for the core PIF study was that at least one parent of the new-born child identified themselves as being of Pacific Island ethnicity and was a permanent resident of New Zealand. South Auckland has the highest number of Pacific Island population in New Zealand [31] from which this cohort was selected. The participants who were healthy were enrolled into this sub-study at the same time as they were recruited into the PIF core study. Consent and assent was sought from children and their mothers before commencing this study. Children who provided assent but had short nails (typically less than 0.05 g (g) of cut material) were excluded. This study was approved by the NZ health and disability ethics committee (NTX/07/05/050).

### 2.2. Heavy Metal Assessment

Toenails were used as a matrix for detecting heavy metals which included chromium (Cr), lead (Pb), cadmium (Cd), copper (Cu), nickel (Ni), and aluminum (Al). Toenail sample collections were conducted in the school setting. Approximately > 50 milligram (mg) of toenail clippings from all toes were collected. These were then placed in a sealed zip-lock polythene bag and each sample was identified with a unique code identification number to ensure anonymity when analyzed. The samples were then stored at room temperature until laboratory analysis.

#### Toenail Sample Laboratory Analysis

There were different steps employed for the chemical analysis.

Step 1: Digestion of toenails: All potential external contaminants in toenails, such as cosmetic treatments and “dirt” were washed prior. The washing procedure involved: (1) five steps using acetone, deionized distilled water (DDW, 18.2 MΩ) (x3) then acetone again; (2) at each washing step enough liquid was added to cover the sample and sonication (ranssonic water bath (T460/H)) for 5–10 min; and (3) decantation. Following the washing procedure, the nail samples were dried overnight at 60 °C in a drying oven (LTE Scientific). Samples that did not have enough toenails were further processed using a higher dilution factor.

Step 2: Chemical Analysis: The washed and processed nail samples was then analyzed in the Agilent 7700 x ICP-MS instrument (Agilent Technology, Santa Clara, CA, USA). The certified reference material (CRM) used were NIST SRM 1643e (National Institute of Standards and Technology, Gaithersburg, MD, USA) and TMDA-54.4 (National Water Research Institute, Canada). The recovery rate range for all the elements was between 76% to 101.3%. All instrumental data for each element (according to the isotope selected) was reported as counts per second. The value was corrected for a reagent blank signal (to correct for any contribution from the digestion procedure) and ratioed with the internal standard isotope value (to correct any instrumental drift or signal enhancement/depression caused by the matrix). Data for the calibration standards was handled in the same manner and an Excel™ calibration curve produced for each element, with ratio signal (y-axis) and concentration of five standards (x-axis), from which the calibration equation was determined for calculation of the unknown toenail sample elemental concentration. The elemental values for each toenail sample were corrected for the dilution factor and the final values used in this data analysis.

### 2.3. Variables

Validated and reliable questionnaires were administered at the nine-year phase to both mother and child. These questionnaires were interviewer-administered to collect information on socio–demographic, cultural, environmental, child development, family and household dynamics, lifestyle, and health issues. Participant characteristics and demographic variables which were of interest for this study were included in the analysis.

Demographics: Child’s gender (girls, boys), child’s Pacific ethnicity, including Samoans, Tongan, Cook Island, and Others group (which included European, Māori, and Niuean), as determined by the mother at age two years phase, household income levels (categorized into $0–$20,000, $20,001–$40,000, > $ 40,000), marital status (non-partnered, partnered, defacto, partnered, legally married).

Lifestyle: Household smoking status (yes, no), number of smokers in households. Secondhand smoking (SHS) was assessed by determining households that smoked versus households that did not smoke.

Health issues: General health (poor, very good), allergies (yes, no), asthma (yes, no).

Weight of children: The average weight and height of children were calculated according to the procedures documented in an operation manual from which the International Obesity Task Force (IOTF) [32] criteria was derived. Prior to data collection, equipment was standardized before weight and height data were collected.

### 2.4. Statistical Analysis

All data which were obtained for this sub-study were stored in Microsoft AccessTM and ExcelTM (Microsoft Corporation, Redmond, WA, USA) template. The data was edited, range and consistency checks were performed, and the data was coded for analysis where necessary. Questions with no response received a distinct code and were not included in the analysis. The edited data was then exported to a statistical software using SAS version 9.4. (SAS Institute Inc., Cary, NC, USA). 

Data were expressed as median with interquartile range for continuous variables and categorical variables were presented as counts and proportions. Mean and standard deviation was also calculated for comparison with published values. The Chi-square test was used to test for association across the demographics variables by those who smoked in the household versus those who did not. Because the variables were not distributed normally, nonparametric statistics were used throughout the study. The between-group differences in the elements were analyzed using the Mann–Whitney U test. The Spearman rank test were used to determine correlations between the elements and number of smokers in household. All *p* values were two-tailed, and *p* < 0.05 was considered statistically significant.

## 3. Results

### 3.1. Demographics Characteristics and Smoking Status

The study sample consisted of 278 children within the Pacific Island Families cohort recruited at the nine-year-old phase. Almost a quarter (37%) of household smoked within this study. There were more proportions of Cook Island peoples who smoked (51%) compared to other ethnicities. There were more male children within our sample (58%) than female children (43%) as seen in Table 1. The majority of the children were of Samoan ethnicity (53%) followed by Cook Island (19%), Others group (17%) and then Tongans (10%). More than half of the participants’ household incomes fell within the $20,001–$40,000 range at the nine-year phase. Around 28% of the mothers thought that their children had poor overall health. The majority of their mothers were in a partnered, legally married status (58%) and had a post-secondary qualification (45%). More than half of the children were either obese (42%) or overweight (26%). Only 15% of the children had asthma or allergies (Table 1). Partnered, de facto category had greater smoking prevalence than the non-partnered or partnered, legally married category (sig < 0.05). Significant associations were observed for ethnicity and marital status in relation to SHS. The other demographic variables were not significantly associated with smoking household.

### 3.2. Household Smoking and Heavy Metal Concentrations

Table 2 indicates smoking households versus non-smoking households and heavy metal concentrations. Even though the levels of Cr, Pb, Ni, and Al were slightly higher in the smoking household compared to those from non-smoking household, it was found to be not statistically significant (Table 2). Cu was found to be higher in the non-smoking household but not significant. 

The heavy metal concentrations were compared with published values by Rodhushkin and Axelsson (2000)) [33] as well as the references for concentrations recognized for optimal health [34]. As shown in Table 3, Cr, Pb, Cd, Cu, and Ni were higher in both smoking and non-smoking household groups than the previously published values [33] and the optimal value required for health [34]. With regards to the published values by Rodhushkin and Axelsson [33], all of the heavy metals in this study fell within the minimum and maximum range except Cu, which was much higher. There was no optimal health value for Al and compared to the published values. The Al concentration in this study was much lower.

### 3.3. Heavy Metal Correlations

Correlations between the heavy metals and the number of smoking household suggest that there is no strong significant association but there was a strong positive correlation between Pb and Cu and moderate positive association in Al with Pb, Cu and Ni (Table 4).

## 4. Discussion

This is the first study that explored any relationship between SHS and heavy metal concentrations in nine-year-old Pacific Island children living in Auckland, NZ. High levels of heavy metals were observed in these children that exceeded the value required for optimal health, although no significant difference in heavy metals with regards to SHS was observed. Additionally, exposure to SHS was associated with children’s ethnicity and parental marital status, but not with household income levels or maternal education within this study. An interesting correlation was observed between Pb and Cu within this study.

### 4.1. Secondhand Smoking

Tobacco smoke is known to contain toxic metals that people are exposed to when smoking or near smokers [35]. Smokers and SHS are known to have higher levels of heavy metals than nonsmokers [36], however, this was not the case in this study. High heavy metal concentrations in children with SHS exposure are understandable, however, the non-SHS children in this sample may have been exposed to a fairly new phenomenon called thirdhand smoke (THS), due to their high concentrations of heavy metals. THS is made up of tobacco gasses and particulates that settle on surfaces and dust for months after someone has smoked a cigarette [37,38]. THS particulates and gasses are also known to reemit into the gas phase or react with other atmospheric materials, releasing secondary contaminants [38]. A recent animal study found that short-term exposure to THS in mice made them more likely to get lung cancer than non-exposed mice [39]. New knowledge on tobacco use has now been shown to be detrimental for both smokers and non-smokers due to the SHS and THS effects [40]. Future studies should focus on THS within Pacific Island peoples so that policies and guidelines can be provided. 

### 4.2. Smoking Demographics

In the Pacific Island Family cohort, at the 4 year phase, 24% of the mothers and 41% of the fathers smoked tobacco [30] which possibly exposes these children not just to SHS but also THS. In the current sub-study at the nine-year phase, almost a quarter of the households smoked tobacco cigarettes. This is not surprising, as a NZ national survey reported that the Pacific Island people have higher smoking rates than their NZ Europeans counterparts [41]. Furthermore, smoking is a major cause of death within the Pacific population in NZ [29]. It is more likely that the non-SHS exposed children within this study may have been exposed to THS if any of their family members smoked or they went to houses of families that may have smoked, exposing them to THS. This was not captured in the current study. Further research is needed within the Pacific Island community to assess THS exposure and its impacts on children.

Within this study, it was observed that majority of the household smokers were of Cook Island ethnicity (51%). This is similar to a study that showed higher rates of smoking amongst Cook Island peoples than other Pacific ethnicities [42]. According to Nosa et.al. [42], the high smoking prevalence could be due to higher acculturation than other Pacific Island ethnic groups or a reflection of the liberal tobacco policy in the Cook Islands [42]. An Australian study found that smoking is normalized within the Arabic-speaking ethnicities in Western Sydney, and this has raised a concern for their children and their wives who may have been exposed to SHS [43]. 

Marital status had an influence on the smoking status in the current study. This is in accordance with another study in which those that were married or co-habited smoked less frequently [44]. Another study also found similar results whereby living without a spouse increased daily smoking rates among both males and females [45]. Other findings suggest that marital status provides a protective effect on smoking in women more than in men [46]. A recent study confirmed the above findings that smoking prevalence was varied by marital status as well as race/ethnicity [47]. 

### 4.3. Heavy Metal Concentrations in Children

The overall concentrations of heavy metals (Cr, Pb, Cd, Cu, and Ni) in all the children were higher than previously published values [33] and the optimal value required for health [34]. However, this study did not show any differences in heavy metal concentrations between household smokers versus non-household smokers. The reason could be that other children within the non-household smoking group may have been exposed via other channels like THS or being around people outside the homes that smoke or outdoors. Similarly, a US National Health and Nutrition Examination Survey for 2013–2014 found that that nonsmokers can be exposed to tobacco smoke outdoors and not just necessarily indoors [48]. Similarly, a study in Portugal found that 19% of children living with parents that do not smoke are still exposed to tobacco smoke [49]. A Spanish study found that 3.9% of children were exposed to SHS in their leisure time and 33.2% while using private or public transportation [50]. A recent study did not find any differences in hair heavy metals between children that were exposed to tobacco smoke exposure versus non exposure [51]. Other ways of tobacco exposures were not measured in this study. Further research is required to understand the harmful effects and toxic metal body burden due to SHS and THS within Pacific Island peoples. 

Cadmium is a well-known toxic metal has an effect on the kidneys and respiratory and skeletal systems [52]. According to Afridi et al. [53], cadmium exposure can lead to hypertension due to the effects this has on the kidneys, causing water imbalance and salt retention. Furthermore, cadmium has a half-life of 10–35 years and accumulates to cause increased excretion of low molecular weight proteins in the urine, which is generally irreversible [54]. Cd also causes disturbances in calcium metabolism and forms kidney stones [54]. Pacific Island people generally have higher rates of chronic kidney disease and end stage renal failure compared with the NZ European population [55]. However, Cd and chronic disease risk within Pacific people is not yet well understood. More follow-up research is required to assess Cd levels within Pacific people, particularly children. It has been observed that Pacific children are low in calcium and zinc levels [56,57] and this in turn can result in higher absorption of Cd and Pb, as studies have reported [58,59]. Within this sample, all the children had high Pb concentrations compared to the optimal required level. Pb has been linked to cognitive and behavioral problems not just in children, but it has shown to carry on into adulthood [60]. A recent follow-up study within the NZ Dunedin Multidisciplinary Health and Development Study has reported that childhood lead exposure has long-term consequences for adult mental health and personality [61]. However, the NZ Dunedin Multidisciplinary Health and Development Study was conducted only amongst the European community [61]. SHS was not associated with Pb in these children, but exposure could have been from living close to motorways or the children could have been in exposed to SHS outside their families or THS. 

Cu is essential for the human body, but excess exposure can cause adverse effects [62]. As children from both smoking households and nonsmoking households had higher Cu levels. Exposures could be from Cu cookware, copper water pipes, or Cu-containing fungicides, however, this was not measured in this study. Furthermore, THS may be the contributor to Cu exposure. Ni is recognized as carcinogenic, which can not only cause lung cancer and nasal cancer, but has also been known to cause respiratory effects [63]. However, THS may also be a contributing factor to the higher Ni concentrations within the current study. Whilst Cr is required in humans at trace amounts, different forms of Cr cause different health effects, for example, Cr (VI) has carcinogenic, mutagenic, and genotoxic effects [64]. Chronic Cr exposure has been detected in smokers [65]. A Tunisian study reported that smokers had higher Cr and Ni levels than controls and their study suggested a possible role of Cr and Ni in developing head and neck cancer [66]. However, this was in the adult population, not children. Al is a neurotoxic element that has been linked to neurodegenerative disorders, such as Alzheimer’s disease [67]. The findings from a meta-analysis suggests that Al exposure is associated with the risk of Alzheimer’s [68]. In animal studies, Al exposure has been linked to learning deficits in animals [69,70]. However, the molecular mechanism underlying Al exposure on learning and memory is still not very clear [68], especially in children. A statistically significant correlation was seen between Pb and Cu in this study. A study found that elevated Pb and Cu levels are correlated with leukocyte counts and iron parameters in adolescents [59]. This new correlation needs to be explored further to understand the antagonistic or synergistic effects Pb and Cu has in cells especially in children. 

### 4.4. Limitations and Strengths

This study should be interpreted with caution, as this is a cross-sectional study. Therefore, the causality between SHS and demographic and outcome variables cannot be identified. Additionally, we did not use any monitoring devices for this study, and so we do not know where the exact exposure source came from.

The reasonable sample size and valid/reliable methods for the heavy metal analysis of toenail samples were the studies strengths. In terms of the direct and sensitive measurement of biological material, toenails were used, which was a non-invasive and more valid method than other indirect measures such as dietary questionnaires or exposure to pollutants using questionnaires. This prevents Type II error (false negative finding) from occurring.

## 5. Conclusions

In conclusion, the present study demonstrated the influence of ethnicity and marital status on exposure to SHS in children. Furthermore, the prevalence of smoking was higher in Cook Island people than other ethnic groups. The most important findings were that heavy metal concentrations were higher than the optimal value in both households that smoked and those households that did not smoke, due to being exposed to tobacco smoke by other means. Further research is required to identify exposure pathways of exposures to tobacco smoke such as THS in Pacific Island children so that politicians and policy makers can provide appropriate guidelines to reduce these exposures.

## Figures and Tables

**Table 1:** Demographics status of children by smoking household.

Demographics	Smoking Household			
	No	Yes	Total	*p*-value
**Gender**				
Male	95 (59.7%)	64 (40.3%)	160 (57.6%)	0.204
Female	78 (67.2%)	38 (32.8%)	118 (42.5%)	
**Ethnicity**				
Samoan	102 (69.9%)	44 (30.1%)	148 (53.2%)	0.048
Tongan	16 57.1%)	12 (42.9%)	29 (10.4%)	
Cook Island	26 49.1%)	27 (50.9%)	53 (19.1%)	
Other *	29 (60.4%)	19 (39.6%)	48 (17.3%)	
Obesity **				
Normal	54 (60.7%)	35 (39.3%)	90 (32.6%)	0.866
Overweight	46 (64.8%)	25 (35.2%)	71 (25.7%)	
Obese	71 (62.8%)	42 (37.2%)	115 (41.7%)	
**General Health**				
Poor	42 (56%)	33 (44%)	75 (27.8%)	0.142
Very good	128 (65.6%)	67 (34.4%)	195 (72.2%)	
**Maternal Education**				
None or secondary	98 (64.5%)	54 (35.5%)	153 (55%)	0.55
Post-school qualification	75 (61%)	48 (39%)	125 (45%)	
**Income category**				
$0–$20,000	54 (59.3%)	37 (40.7%)	93 (34.7%)	0.408
$20,001–$40,000	94 (65.7%)	49 (34.3%)	143 (53.4%)	
>$40,000	17 (54.8%)	14 (45.2%)	32 (11.9%)	
**Allergies**				
No	143 (61.9%)	88 (38.1%)	234 (84.8%)	0.557
Yes	28 (66.7%)	14 (33.3%)	42 (15.2%)	
**Asthma**				
No	142 (61.7%)	88 (38.3%)	233 (84.7%)	0.54
Yes	28 (66.7%)	14 (33.3%)	42 (15.3%)	
**Maternal marital status**				
Non partnered	55 (72.4%)	21 (27.6%)	76 (27.9%)	0.022
Partnered, defacto	18 (46.2%)	21 (53.9%)	39 (14.3%)	
Partnered, legally married	99 (63.1%)	58 (36.9%)	157 (57.7%)	

* European, Māori, and Niuean; ** International obesity task force.

**Table 2:** Median with interquartile range (IQR) of toenail heavy metals (µg/g) by smoking household.

	People Smoking in the Household		
Metals	No; Median (IQR)	Yes; Median (IQR)	*p*-Value
Cr	0.68 (0.44–1.06)	0.7 (0.51–1)	0.701
Pb	0.48 (0.13–1.03)	0.55 (0.19–1.23)	0.304
Cd	0.18 (0.11–0.28)	0.18 (0.12–0.31)	0.45
Cu	17.28 (13.31–22.78)	16.66 (13.39–21.13)	0.784
Ni	0.32 (0.19–0.6)	0.35 (0.18–1.11)	0.32
Al	5.29 (3.78–9.23)	6.28 (4.09–11.4)	0.213

**Table 3:** Comparison of toenail heavy metals (µg/g) with recognized published values.

People Smoking in the Household				
Metals	No Mean (SD)	Yes Mean (SD)	Optimal Health ^a^ (Mean)	Published Min–Max ^b^ (Range)
Cr	1.15 (1.6)	0.9 (0.68)	0.2	0.90–9.7
Pb	0.75 (0.9)	1.05 (1.82)	<1.0	0.04–240
Cd	0.21 (0.12)	0.22 (0.13)	<0.05	0.03–1.9
Cu	19.31 (10.88)	19.26 (10.3)	8.5	9–81
Ni	0.83 (1.62)	1.54 (7.12)	1.5	6
Al	7.64 (6.05)	8.74 (7.03)	-	37.5

^a^ Optimal health (Ward 2008); ^b^ Rodhushkin and Axelsson (2000).

**Table 4:** Correlations between the toenail heavy metal concentrations (µg/g) and number of smokers in household.

Spearman Correlation *p*-Value	Number of Household Smoking	Chromium (Cr)	Lead (Pb)	Cadmium (Cd)	Copper (Cu)	Nickel (Ni)	Aluminum (Al)
Number of household smoking	1	0.02344	0.04308	0.02616	0.00297	0.06354	0.08178
		0.6987	0.4768	0.6658	0.9609	0.2937	0.1763
Cr		1	0.00982	−0.1359	−0.03769	−0.0235	−0.01618
			0.8705	0.0235	0.5314	0.6965	0.7882
Pb			1	−0.0442	0.60024	0.21718	0.39401
				0.4633	<0.0001	0.0003	<0.0001
Cd				1	−0.03131	−0.03736	−0.00513
					0.6032	0.535	0.9321
Cu					1	0.24464	0.38339
						<0.0001	<0.0001
Ni						1	0.29339
							<0.0001
Al							1
						
